# The effect of supplementary light on the photosynthetic apparatus of strawberry plants under salinity and alkalinity stress

**DOI:** 10.1038/s41598-022-17377-8

**Published:** 2022-08-02

**Authors:** Mohammad Reza Malekzadeh Shamsabad, Majid Esmaeilizadeh, Hamid Reza Roosta, Mohammad Reza Dehghani, Piotr Dąbrowski, Hazem M. Kalaji

**Affiliations:** 1 Department of Horticultural Sciences, Faculty of Agriculture, Vali-e-Asr University of Rafsanjan, Kerman, 7718817111 Iran; 2 Department of Horticultural Sciences, Faculty of Agriculture and Natural Resources, Arak University, Arak, 38156-8-8349 Iran; 3 Department of Genetics and Plant Production, Faculty of Agriculture, Vali-e-Asr University of Rafsanjan, Kerman, 7718817111 Iran; 4 Department of Environmental Development, Institute of Environmental Engineering, Warsaw University of Life Sciences-SGGW, Nowoursynowska Str. 159, 02-776 Warsaw, Poland; 5 Department of Plant Physiology, Institute of Biology, Warsaw, University of Life Science, 159 Nowoursynowska St., 02-776 Warsaw, Poland; 6 Institute of Technology and Life Sciences-National Research Institute, Falenty, Al. Hrabska 3, 05-090 Raszyn, Poland

**Keywords:** Light responses, Photosystem I, Photosystem II, Abiotic

## Abstract

Considering the destructive effect of stresses on the photosynthetic apparatus of plants and the important role of light in photosynthesis, we investigated the effect of complementary light on the photosynthetic apparatus under salinity and alkalinity stress conditions. Light-emitting diodes (LEDs) in monochromatic blue (460 nm), monochromatic red (660 nm), dichromatic blue/red (1:3), white/yellow (400–700 nm) at 200 μmol m^−2^ S^−1^, and without LED treatment were used. The stress treatments were in three stages: Control (no stress), Alkalinity (40 mM NaHCO_3_), and Salinity (80 mM NaCl). Our results showed that salinity and alkaline stress reduced CO_2_ assimilation by 62.64% and 40.81%, respectively, compared to the control treatment. The blue light spectrum had the highest increase in water use efficiency (54%) compared to the treatment without supplementary light. Under salinity and alkalinity stress, L, K, and H bands increased and G bands decreased compared to the control treatment, with blue/red light causing the highest increase in L and K bands under both stress conditions. In salinity and alkalinity stress, white/yellow and blue/red spectra caused the highest increase in H bands. Complementary light spectra increased the G band compared to the treatment without complementary light. There was a significant decrease in power indices and quantum power parameters due to salt and alkalinity stress. The use of light spectra, especially blue, red, and blue/red light, increased these parameters compared with treatment without complementary light. Different light spectra have different effects on the photosynthetic apparatus of plants. It can be concluded that using red, blue spectra and their combination can increase the resistance of plants to stress conditions and be adopted as a strategy in planting plants under stress conditions.

## Introduction

Light quality detection and measurement are essential for plant growth and development^1^. Under natural growth conditions, due to environmental factors such as clouds, rain, and other climatic factors, natural light may not be enough for optimal plant growth. Under these conditions, artificial light sources in the controlled cultivation of plants promote high yield and production of quality products^2^. Light-emitting diodes (LEDs) can also be used as light sources for planting systems and photobiological research. These LEDs have a low mass, small size, long lifetime, and narrow spectral output. The use of light-emitting diodes as light sources in agriculture has been increasing in recent years^3^.

Photosynthetic pigments absorb light energy and convert it into chemical energy, a process in the photosynthetic apparatus. Blue and red spectra play an important role in photosynthesis^4^. Blue and red lights have been shown to induce stomatal opening^5^. An important tool for researching the effects of various stresses on photosynthesis in plants is chlorophyll *a* fluorescence (Chl) kinetics^6^. The photochemical efficiency of plant photosynthesis is represented by fluorescence kinetics. This tool provides important information about the functional and structural properties of the components involved in photosynthetic electron transfer^7^. The use of combined tools (Photosynthetic Efficiency Analysis of plants and Measurement of Plant Gas Exchange) permitted the exploration and description of responses to stress^8^. The JIP test has been successfully used to study the photosynthetic apparatus of various plant species under abiotic stresses^9,10^.

With increasing population and degradation of natural environments, soil salinization has become a serious global problem^11^. Approximately 7% of the world's land (more than 900 million hectares) is at risk of salinity and alkalinity^12^. Salinity and alkaline stresses as two abiotic stresses have severely limited the development of global agriculture. Therefore, the study of plant resistance mechanisms in salinity and alkaline stress conditions is of great practical importance for the development of strategies for tolerance to salinity and alkalinity stresses. It is possible to increase plant tolerance to stress conditions for sustainable agricultural development by using methods^13^. Salinity stress affects the transfer of electrons from the reaction centers (RCs) to the plastoquinone pool. Salinity stress disrupts electron transfer^14^, and adversely affects photosynthetic efficiency^15^. These destructive effects reduce the efficiency of photosynthesis^16^. Salt accumulation in mesophilic cells prevents carbon uptake, thus increasing the internal CO_2_ concentration and ultimately decreasing the stomatal conductance^17^. One of the criteria for studying stress tolerance in plants is the attributes of plant gas exchange^18^. Plant gas exchange properties are one of the most important physiological characteristics that significant changes in these properties occur due to salinity stress^19^. Growth of different strawberry cultivars with alkaline stress decreased due to reduction of chlorophyll content and inhibition of photosynthesis and electron transfer was significantly inhibited, which led to a decrease in photosynthetic yield index^20^. In an experiment, salinity stress reduced chlorophyll content. It reduced vegetative and reproductive growth in the Paros strawberry cultivar, and inhibition of chlorophyll biosynthesis, acceleration of its degradation, and oxidative damage caused by salinity were considered as the main reasons for chlorophyll content reduction^21^.

Much research has been done on the growth response of plants to light quality^22^, and its effect on plant photosynthesis^23^. Hyo et al.^24^ investigated the effect of LED light on strawberries in the greenhouse and growth chamber. They showed that different spectrums of LED lights in various conditions have different effects on the growth and development of strawberry plants. They suggested that growing strawberries in a greenhouse with LED lighting is more efficient than planting in a growth chamber. Much research has been done on the effects of blue and red spectra on plants^25^, but it is not easy to reach a consensus on the effect of an optical spectrum on plant species. Light duration, variety, and environmental stresses such as salinity, alkalinity, drought, and high temperatures also affect plant growth and may alter the effects of light spectra. These studies can also be generalized to environmental stresses (salinity, alkalinity, drought, etc.). The role of different light spectra on the photosynthetic apparatus under stress conditions can be evaluated using chlorophyll fluorescence and gas exchange measurements.

This work aimed to understand better the mechanism of photosynthetic apparatus response to salinity and alkaline stresses under different light spectra. We tried to answer the question of which spectrum or spectra of complementary light can improve the performance of the photosynthetic apparatus under salinity and alkalinity stress conditions. We tried to show how complementary light spectra affect the photosynthetic apparatus of the Paros strawberry cultivar under salinity and alkalinity stress conditions. We hypothesize that the use of complementary light can have a positive effect on the photosynthesis apparatus of strawberry plants. The manipulation of the light spectra used by plants has a significant positive effect on the efficiency of the photosynthesis apparatus in stressed plants. Therefore, selecting the appropriate light spectrum can reduce the destructive effects of salinity and alkalinity stress. We used LEDs as complementary lighting in the greenhouse and under stress conditions. Expanding the use of LEDs as complementary lighting in greenhouses and stress conditions is important for commercial horticulture and plant research.

## Materials and methods

### Plant material and growth conditions

In 2020, the experiment was conducted in the research greenhouse at Vali-e-Asr University of Rafsanjan- Iran (Latitude: 30°21'17.6004'' N, Longitude: 56°0'9.738'' E, Elevation: 1545.924). Bare root plants of strawberry (*Fragaria* × *ananassa* Duch, cv. Paros) were obtained from a nursery in Karaj, Iran. Plants were planted in a pot of 4 l containing cocopeat and perlite (70:30 ratio). Each treatment included three pots, and each pot was an experimental unit in which three plants were planted. Pots with a width of 30 cm were used to plant three plants. Plants were cultivated in the greenhouse with a temperature of 25/15 ± 2 ºC (day/night), a photoperiod of 11.13 h (light/dark), and relative humidity of 50 ± 10%. During this period, plants were fertigated with the Morgan nutrient solution (Morgan 2003) (Electrical Conductivity (EC): 1.4 dS m^−1^ and pH 6.5) (Table 1). Plants were treated with five light levels and two stress levels, including control (without stress), alkalinity (40 mM NaHCO_3_), and salinity (80 mM NaCl). The alkalinity and salinity treatments were applied twenty days after planting. 100 ml of NaHCO_3_ and NaCl were added to each pot to maintain uniform stress for the plants. Stresses continued until the end of the experiment and the completion of data collection.

**Table 1:** The concentration of nutrients used in the nutrient solution of this experiment.

Macronutrients	Concentration (mg L^−1^)	Micronutrients	Concentration (mg L^−1^)
N	128	Fe	5
P	58	Mn	2
K	211	Zn	0.25
Ca	104	B	0.7
Mg	40	Cu	0.07
S	54	Mo	0.05

### Light environment

**Figure 1:**
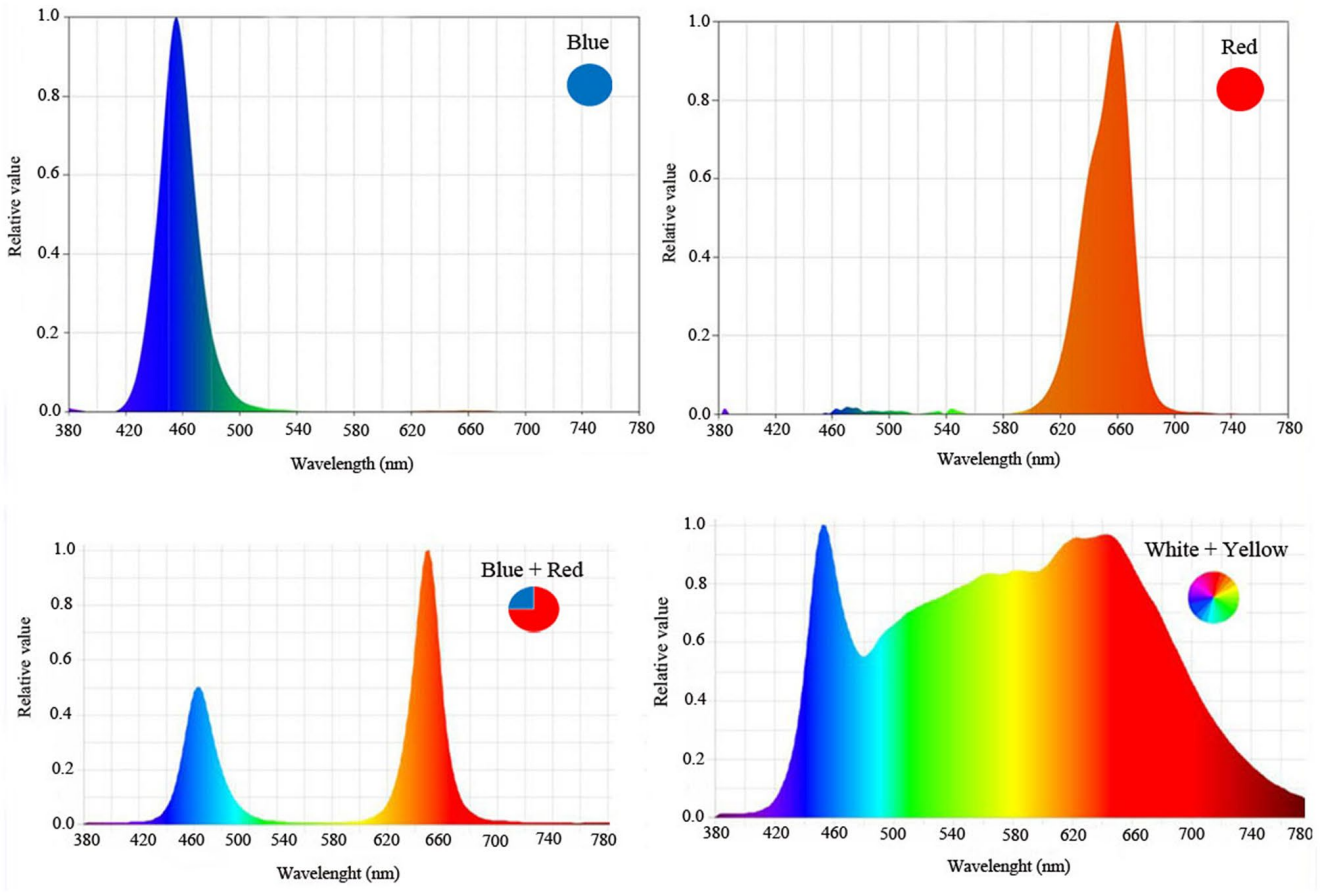
Relative distribution of different spectral LEDs (monochromatic blue, monochromatic red, dichromatic blue/red (1:3), and dichromatic white/yellow (1:1) used during the plant growth period.

Plants were grown under LED systems (length: 100 mm, width: 5 mm, and height: 5 mm) with 24W LED tubes (Parto Roshd Novin Company, Iran grow light, Iran) of different spectral ranges: monochromatic blue (B) (with peak 460 nm), monochromatic red (R) (with peak 660 nm), dichromatic blue/red (1:3), white/yellow (400–700 nm) (Fig. 1) and without LED treatment.

Photon flux density (PPFD) of LED lights was at 200 μmol m^−2^ s^−1^ per 11-h light period. Directly above each plant, LED lighting systems were mounted 30 cm apart.

### Evaluation of chlorophyll fluorescence

We used a portable photosynthetic efficiency analyzer (PEA, Hansatech Inc. Co., UK) sixty days after planting to measure and calculate chlorophyll fluorescence parameters (PEA, Hansatech Inc. Co., UK). For this purpose, fully mature leaves of each pot were adapted to a dark time for 15 min by fixing special tags on each leaf before measurement. Then the sensor cup was mounted on the leaf for calculation. The chlorophyll *a* fluorescence transient was caused by a saturating photon flux density of 3.500 μmol(photon) m^−2^ s^−1^ given by three light-emitting diodes (peak 650 nm) to produce fluorescence curves ranging from F_o_ to F_m_ (F_t_, fluorescence at time t after the onset of actinic illumination; F_o_ = F_30μs_, minimum fluorescence intensity; F_j_ = F_2ms_, fluorescence intensity at the J-step; F_i_ = F_30ms_, fluorescence intensity at the I-step; F_p_ = F_m_, Maximum fluorescence intensity at peak P of OJIP) for all treatments. The PSII parameters obtained from the OJIP transient were evaluated based on the Strasser et al.^14^ methods. Parameters for chlorophyll fluorescence are listed in an Suppl. Appendix Table.

### Leaf gas exchange

Plant gas exchange parameters include net CO_2_ assimilation rate (A, μmol CO_2_ m^−2^ s^−1^), intrinsic water-use efficiency (WUE_i_, μmol CO_2_ mol H_2_O^−1^), stomatal conductance (G_s_, mol H_2_O m^−2^ s^−1^), transpiration (E, mmol H_2_O m^−2^ s^−1^), Sub-stomatal CO_2_ concentration (C_i_, μmol CO_2_ mol^−1^) and, instantaneous carboxylation efficiency (A/C_i_) were measured using a portable photosynthesis system (ADC BioScientific Ltd, Hoddesdon, UK) after 60 days after planting. Around 9:00 AM and 12:00 AM, measurements were performed on completely expanded leaves.

### Experimental design and data analysis

The experiment was factorial, consisting of a randomized, complete design with two factors in three replicates and three single plants per pot. All data were analyzed using SAS software version 9.4 (SAS Institute, Cary, NC, USA. https://www.sas.com/en_us/home.Html). When variance analysis (ANOVA) indicated significant treatment effects, significant mean variations (P < 0.05) were calculated using the LSD Multiple Range Test. Biophysical parameters were determined with "PEA Plus" software version 1.12 (http://www.hansatech-instruments.com). Pearson's correlation coefficient was applied to determine the relationships among the parameters studied. Principal component analysis was performed using MATLAB software version R2015b (https://www.mathworks.com). A correlation plot was drawn with Origin Pro software version 2021 (https://www.originlab.com/2021). The biplots were made using Excel software version 2016 (https://www.microsoft.com)^26^.

### Statement of compliance

The authors confirm that all the experimental research and field studies on strawberry plants, including the collection of plant material, complied with relevant institutional, national, and international guidelines and legislation. Also, obtained licenses for the preparation of Bare root plants of strawberry.

## Results

### Leaf gas exchange analyses

The analysis of ANOVA showed that plant gas exchange parameters were significantly affected by different light spectra, stress, and their interaction effects. Net CO_2_ assimilation (A) of plants was influenced considerably by salinity and alkalinity stress and different light spectra. Net CO_2_ assimilation decreased under salinity and alkalinity stress compared to the control. Under salinity stress, blue/red and red light had the greatest effect on increasing CO_2_ assimilation under stress conditions and had the lowest percentage of reduction compared to the control treatment. Blue/red light has the largest level of CO_2_ assimilation under alkalinity stress. There was the least decrease in CO_2_ assimilation in blue light relative to the control treatment, and the highest percentage reduction was found without LED treatment (Table 2).

**Table 2:** Effect of light spectra and stress on leaf gas exchange parameters of strawberry cv. Paros.

Light sources	Stress	A (μmol CO_2_ m^−2^ s^−1^)	E (mmol H_2_O m^−2^ s^−1^)	G_s_ (mol H_2_O m^2^s^−1^)	C_i_ (μmol CO_2_ mol^−1^)	WUE_i_ (μmol CO_2_ mol H_2_O^−1^)	A/C_i_
Blue	Control	20 ± 0.6^a^	9.91 ± 0.29^bc^	0.280 ± 0.001^b^	167 ± 7.8^d^	5.2 ± 2.0^a^	0.12 ± 0.005^b^
	Salinity	5.99 ± 0.29^i^	8.5 ± 0.63^de^	0.114 ± 0.003^hi^	255 ± 8^b^	2.5 ± 0.36^bc^	0.023 ± 0.004^g^
	Alkalinity	12.5 ± 0.37^f^	4.97 ± 0.49^gh^	0.190 ± 0.005^d^	207 ± 2.9^c^	3.1 ± 0.84^b^	0.06 ± 0.002^de^
Red	Control	18 ± 0.33^c^	13.2 ± 0.13^a^	0.280 ± 0.005^b^	93 ± 1.4^f^	1.9 ± 0.3^c^	0.192 ± 0.008^a^
	Salinity	8.32 ± 0.15^h^	5.9 ± 0.42^g^	0.160 ± 0.005^f^	218 ± 12^c^	1.2 ± 0.1^d^	0.038 ± 0.002^f^
	Alkalinity	9.96 ± 0.13^g^	8.15 ± 0.39^def^	0.166 ± 0.008^ef^	164 ± 5.4^d^	1.8 ± 0.3^c^	0.06 ± 0.001^de^
Blue/Red	Control	27.6 ± 0.16^a^	8.92 ± 0.25^cd^	0.363 ± 0.008^a^	142 ± 3^e^	2.3 ± 0.5^bc^	0.194 ± 0.003^a^
	Salinity	12.7 ± 0.68^f^	7.17 ± 0.26^f^	0.183 ± 0.003^d^	222 ± 3.5^c^	1.2 ± 0.1^d^	0.057 ± 0.003^e^
	Alkalinity	14.3 ± 0.2^e^	7.86 ± 0.52^ef^	0.180 ± 0.005^de^	166 ± 3.9^d^	1.8 ± 0.1^c^	0.086 ± 0.002^c^
white/yellow	Control	16.9 ± 0.34^d^	10.1 ± 0.45^b^	0.226 ± 0.008^c^	209 ± 0.57^c^	2.0 ± 0.1^cd^	0.08 ± 0.001^c^
	Salinity	5.89 ± 0.11^i^	5.59 ± 0.12^gh^	0.116 ± 0.003^h^	255 ± 1.2^b^	0.9 ± 0.3^e^	0.023 ± 0.004^g^
	Alkalinity	8.47 ± 0.51^h^	7.24 ± 0.36^f^	0.143 ± 0.003^g^	215 ± 2.3^c^	1.6 ± 0.4^cd^	0.039 ± 0.002^f^
Ambient	Control	14.4 ± 0.24^e^	7.42 ± 0.31^f^	0.240 ± 0.005^c^	222 ± 1.1^c^	3.3 ± 0.3^b^	0.064 ± 0.001^d^
	Salinity	4.94 ± 0.21^j^	3.88 ± 0.03^i^	0.100 ± 0.001^i^	278 ± 1.4^a^	1.8 ± 0.3^cd^	0.017 ± 0.007^g^
	Alkalinity	6.2 ± 0.19^i^	4.64 ± 0.12^hi^	0.120 ± 0.001^h^	272 ± 10^a^	1.9 ± 0.4^cd^	0.022 ± 0.005^g^
Significance	Light (L)	***	***	***	***	***	***
	Stress (S)	***	***	***	***	***	***
	L × S	***	***	***	***	**	***

Values are means ± SE of three replicates. Bars with different letters show significant differences at P ≤ 0.05 (LSD). Significance according to ANOVA, ns, ∗ , ∗∗ , ∗∗ ∗ , nonsignificant and significant P ≤ 0.05, 0.01, 0.001, respectively. Control (non-stress), salinity (80 mM NaCl) and alkalinity (40 mM NaHCO3). Photon flux density for the complement spectrum (PPFD) 200 ± 10 mmol m^–2^ s^–1^. SAS software version 9.4 was used for data analysis (https://www.sas.com/ en_ us/home.Html).

A comparison of the mean interactions of different light spectra and stress on plant gas exchange parameters of strawberry plants showed that transpiration rate (E) and stomatal conductance (G_s_) decreased under salinity and alkalinity stress. The lowest percentage reduction transpiration rate (E) under salinity stress was observed in blue and blue/red light with 16% and 19% reduction compared to the control treatment, respectively. Under alkaline stress, the lowest percentage of transpiration (E) was observed in blue/red light with a decrease of 11% compared to the control treatment. The red light had the greatest effect on the Gs parameter under salinity conditions and caused the least decrease (42%) compared to the control treatment. Under alkalinity stress, blue light had the greatest effect on the E parameter.

Internal CO_2_ (C_i_) concentrations under stress conditions were significantly affected by different light spectra. Under stress conditions, this parameter increased, and the application of light treatment decreased Ci. Blue/red light increased the instantaneous carboxylation efficiency (A/C_i_) parameter compared to other light spectra.

Salinity and alkalinity stress reduced water use efficiency (WUE_i_) compared to the control treatment. Under all stress conditions, blue/red light significantly increased WUEi. The lowest WUEi was observed in the without supplementary light treatment.

### Prompt chlorophyll *a* fluorescence

**Figure 2:**
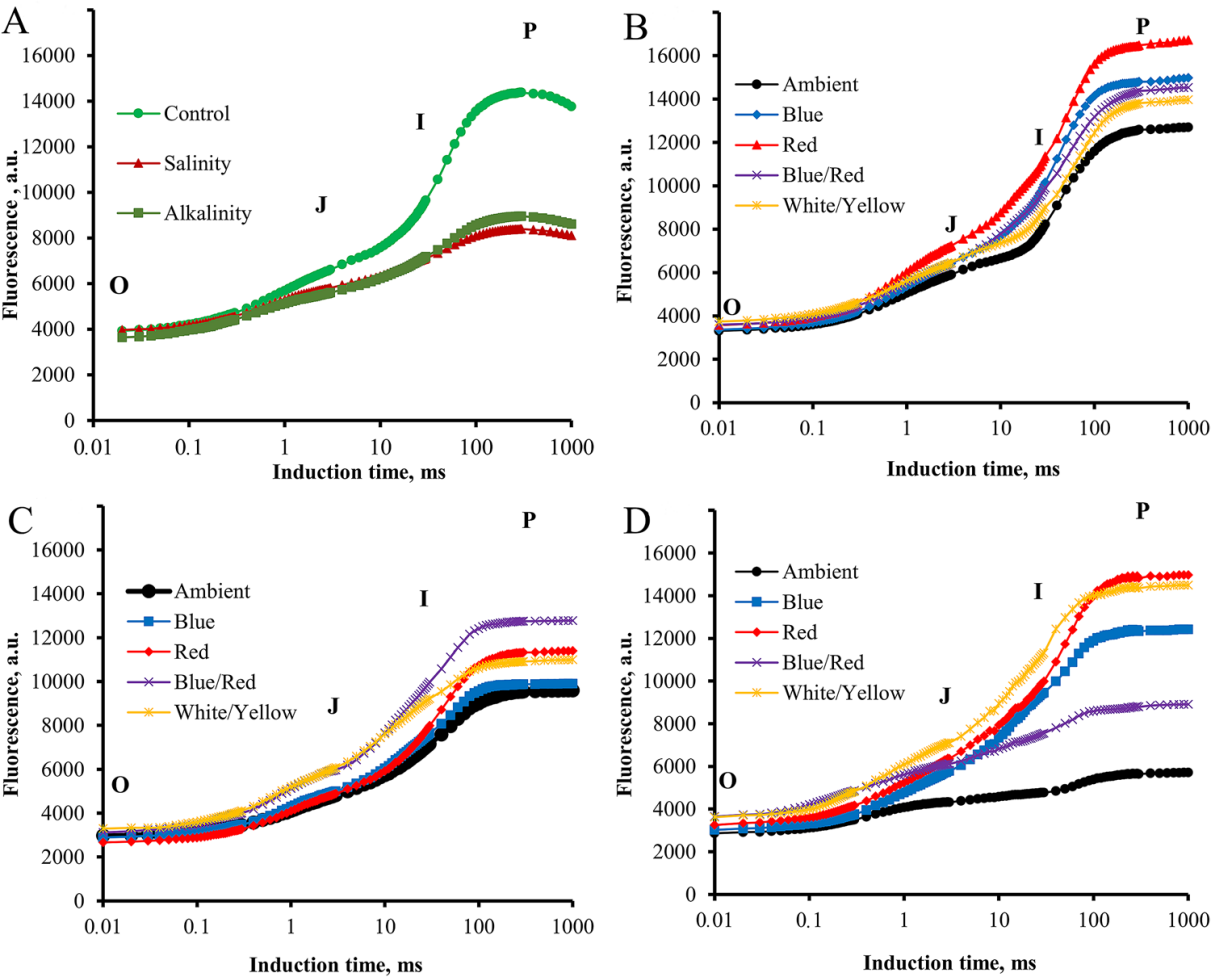
Induction curves of chlorophyll a fluorescence of strawberry cv. Paros. (**A**) In ambient light (control, salt stress, alkalinity stress); (**B**) non-stressed in different light spectra (ambient, blue, red, blue/red, white/yellow); (**C**) under salt stress in different light spectra (ambient, blue, red, blue/red, white/yellow); (**D**) under alkalinity stress in different light spectra (ambient, blue, red, blue/red, white/yellow). Biophysical parameters were determined with "PEA Plus" software version 1.12 (http://www.hansatech-instruments.com). The graphs were drawn using Microsoft Excel version 2016 (https://www.microsoft.com).

According to the results, stress and light spectrum had a significant effect on the fluorescent transients. Salinity and alkalinity stress reduced the fluorescent transients compared to non-stress conditions. Different light spectra had a positive effect on the fluorescence curve. Under salinity stress, the blue/red spectrum had a significant effect on increasing the fluorescence curve, especially at points I and P compared to other light spectra. Whereas under alkaline stress conditions, red and white/yellow light had the most significant effect on increasing the fluorescent transients. Blue and blue/red light also caused a significant increase in the fluorescent transients compared to the treatment without supplementary light (Fig. 2).

### Chlorophyll fluorescent transients and calculated curves

**Figure 3:**
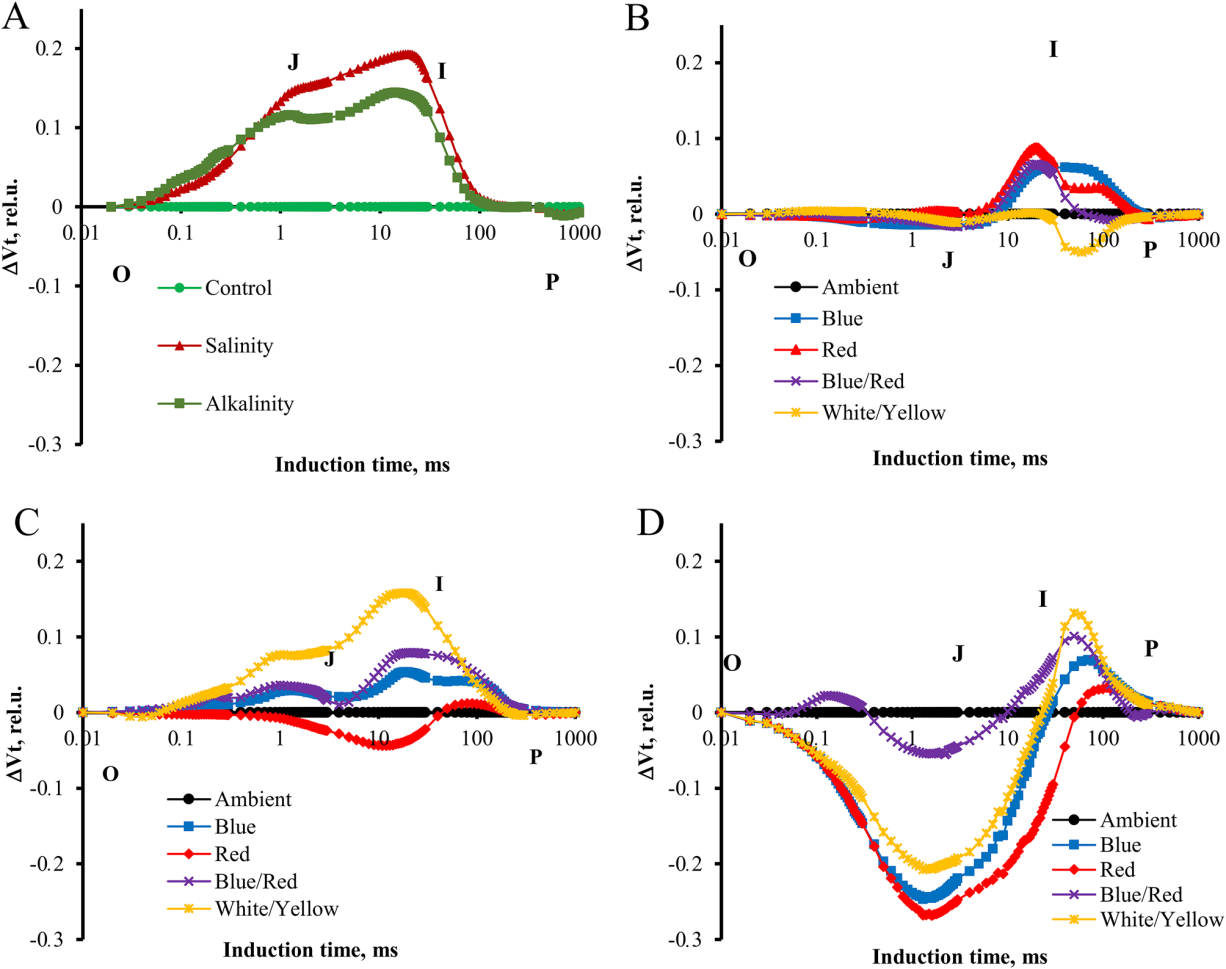
Differential curves of ΔV_t_ of chlorophyll a fluorescence of strawberry cv. Paros. (**A**) In ambient light (control, salt stress, alkalinity stress); (**B**) non-stressed in different light spectra (ambient, blue, red, blue/red, white/yellow); (**C**) under salt stress in different light spectra (ambient, blue, red, blue/red, white/yellow); (**D**) under alkalinity stress in different light spectra (ambient, blue, red, blue/red, white/yellow). Biophysical parameters were determined with "PEA Plus" software version 1.12 (http://www.hansatech-instruments.com). The graphs were drawn using Microsoft Excel version 2016 (https://www.microsoft.com).

To help visualize the influence of the stress and light spectra on the transient dynamics, the curves were plotted as relative variable fluorescence, Vt = (F_t_ − F_O_)/(F_M_ − F_O_)^14^. The changes in the OJIP fluorescence were calculated by subtracting the values of the fluorescence (O–P) recorded in plants under stress from those recorded for control plants. We observed significant changes in the prompt fluorescence of plants under stress and different light spectra at the J (V_J_) and I (V_I_) steps. The characteristics of OJIP fluorescence transients recorded in strawberry plants under different light spectra differed from those recorded without LED treatment. The results showed that the relative variable fluorescence curve in plants under salinity and alkalinity stress was higher than in control plants. Also, salinity stress was higher than alkalinity stress (Fig. 3).

To do a complete analysis of the different light spectra and stress-induced shifts in the OJIP curve, the differential curves were plotted separately for the main band that occurred during the transient O–P: The L, K, H, and G bands. The Curves for these bands were plotted by subtracting the normalized fluorescence values (between O and K, O and J, J and I, and I and P, respectively) observed in control plants from those recorded in plants under different light spectrum and stress conditions.

**Figure 4:**
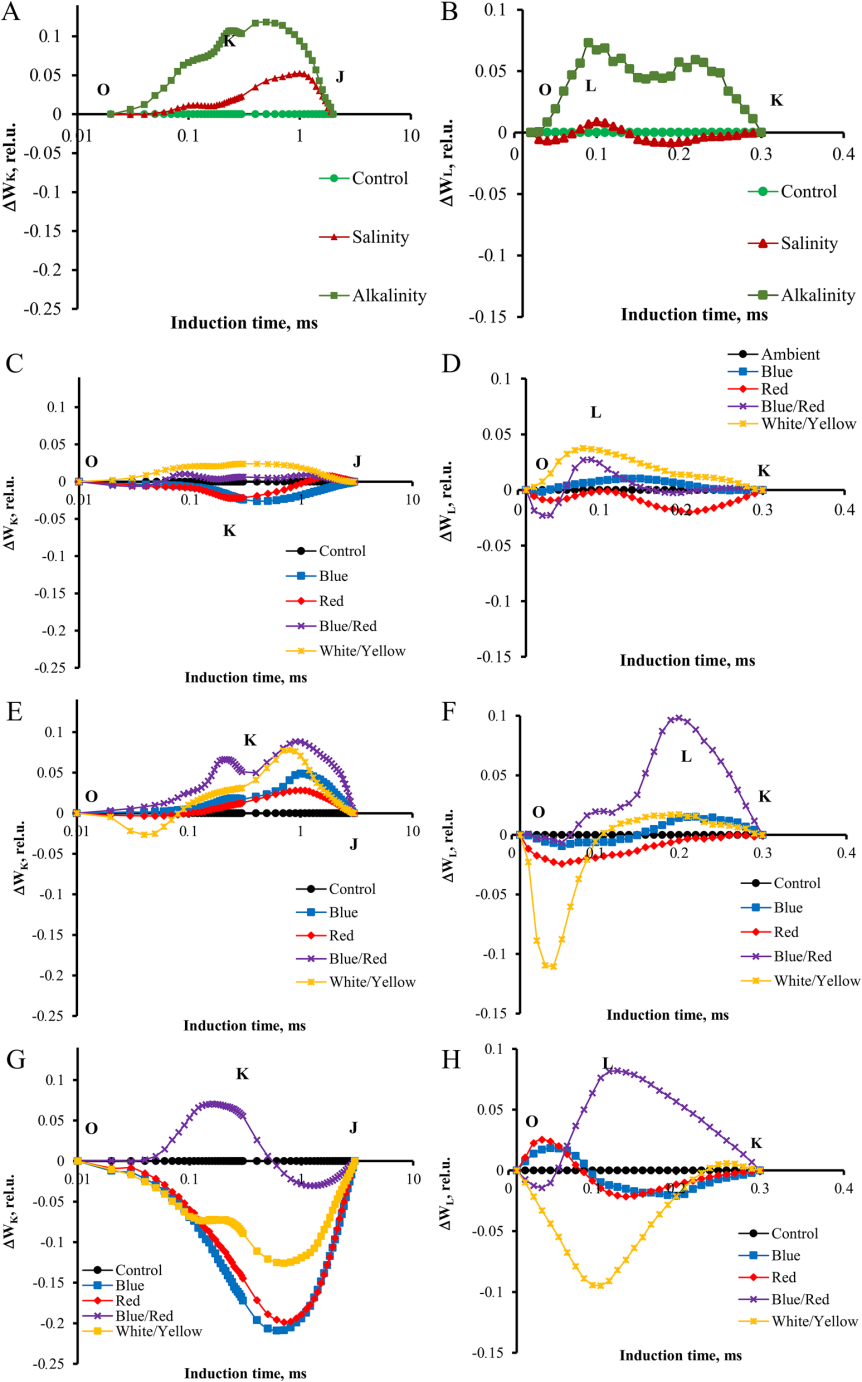
Differential curves of ΔW_K_ and ΔW_L_ of chlorophyll a fluorescence of strawberry cv. Paros. (**A,B**) In ambient light (control, salt stress, alkalinity stress); (**C,D**) non-stressed in different light spectra (ambient, blue, red, blue/red, white/yellow); (**E,F**) under salt stress in different light spectra (ambient, blue, red, blue/red, white/yellow); (**G,H**) under alkalinity stress in different light spectra (ambient, blue, red, blue/red, white/yellow). Biophysical parameters were determined with "PEA Plus" software version 1.12 (http://www.hansatech-instruments.com). The graphs were drawn using Microsoft Excel version 2016 (https://www.microsoft.com).

In ambient light, significant changes in the K band were observed under the influence of alkaline and salt stress caused smaller but also visible changes (Fig. 4). Alkaline stress also caused changes during the L band, which was not observed in the plants treated with salt. In non-stressed plants under the influence of different light conditions, no significant differences were observed in both bands. Under the influence of salt stress, both bands had the highest course in blue/red light and the lowest in red and blue light. Under alkaline stress, both bands had a higher course under blue/red light, while under the other light conditions, the curves had the same course as in ambient.

**Figure 5:**
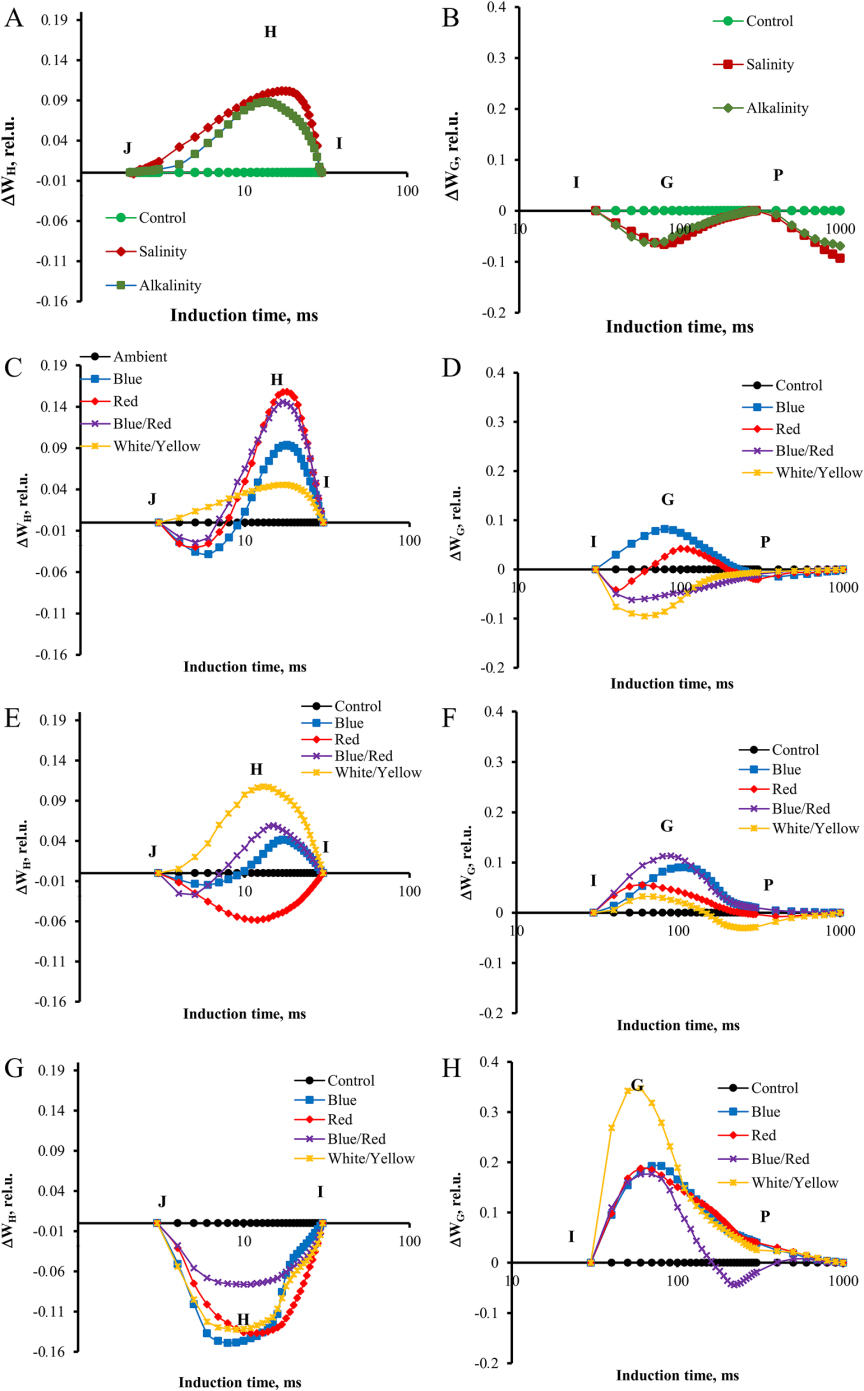
Differential curves of ΔW_H_ and ΔW_G_ of chlorophyll a fluorescence of strawberry cv. Paros. (**A,B**) In ambient light (control, salt stress, alkalinity stress); (**C,D**) non-stressed in different light spectra (ambient, blue, red, blue/red, white/yellow); (**E,F**) under salt stress in different light spectra (ambient, blue, red, blue/red, white/yellow); (**G,H**) under alkalinity stress in different light spectra (ambient, blue, red, blue/red, white/yellow). Biophysical parameters were determined with "PEA Plus" software version 1.12 (http://www.hansatech-instruments.com). The graphs were drawn using Microsoft Excel version 2016 (https://www.microsoft.com).

Both stresses caused a change in the H and G bands (Fig. 5). However, the H band increased, but the G band decreased compared to the control (non-stressed plants). In the ambient treatment, in non-stressed plants, red and blue/red spectra, and salt-treated plants, white/yellow spectra significantly increased the H band. Under alkaline stress, the blue/red spectrum increased the H band more than other light spectra. In the control treatment, the blue and red spectra increased the G band compared to the treatment without supplementary light. Under salinity stress, blue/red and blue light could significantly increase the G band, and under alkaline stress, white/yellow light had the greatest effect on this band (Fig. 5).

### JIP-test parameters, calculated from chlorophyll fluorescence transients

**Figure 6:**
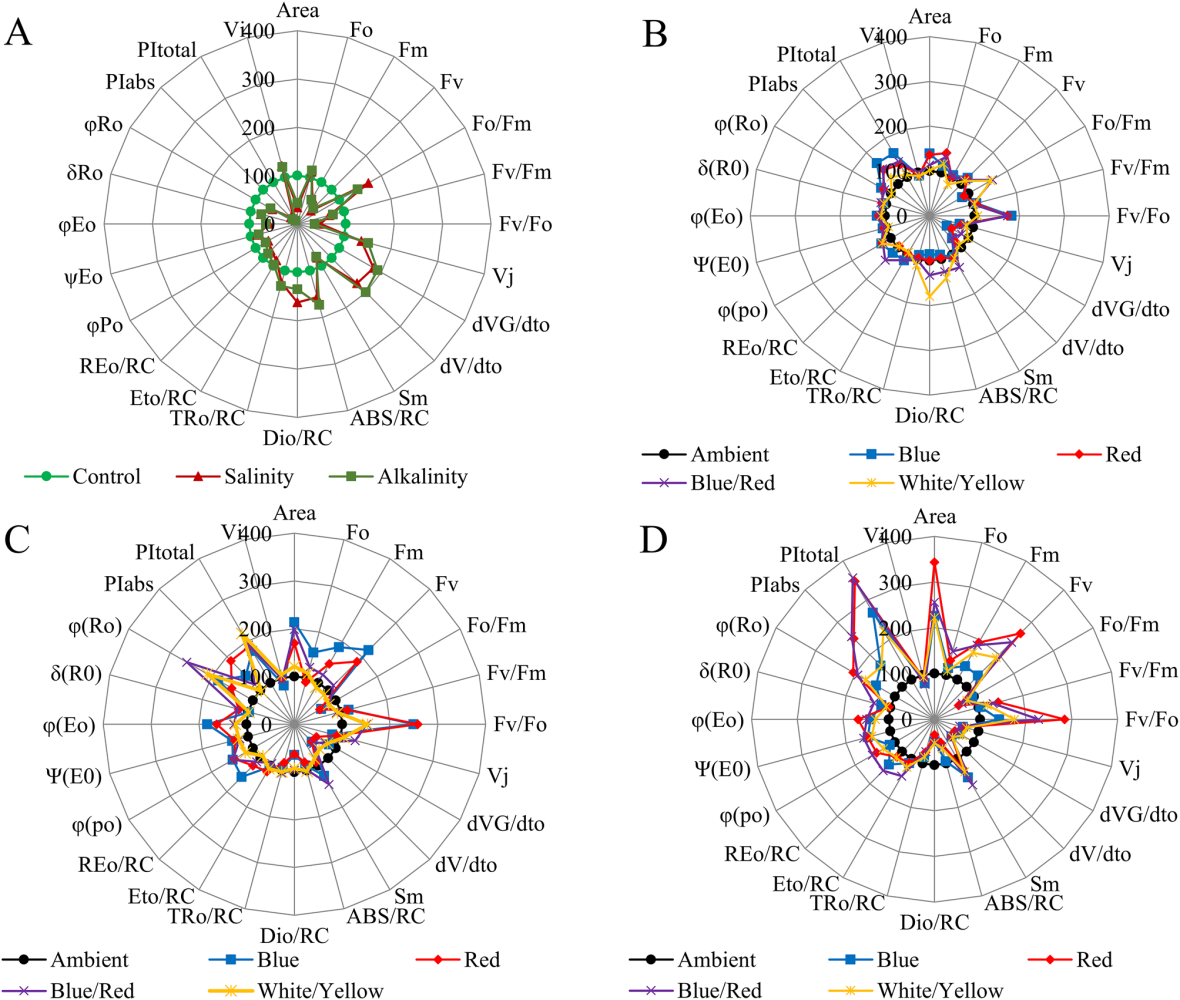
JIP-test parameters normalized on radar plots. (**A**) The effect of ambient light conditions on non-stressed plants; (**B**) the effect of salt stress at different light conditions; (**C**) the effect of alkalinity stress at different light conditions; (**D**) the effects of salinity and alkaline stress at ambient light conditions. Biophysical parameters were determined with "PEA Plus" software version 1.12 (http://www.hansatech-instruments.com). The graphs were drawn using Microsoft Excel version 2016 (https://www.microsoft.com).

OJIP transients have been translated into biophysical parameters: the basic parameters derived from the extracted data, normalized data, specific energy fluxes (per active PSII reaction center), performance indexes, quantum yield for primary photochemistry, and slopes and integrals (Strasser et al. 2010). The values of the measured parameters have been normalized to those of the control plants. On radar plots, the deviation of the activity pattern of plants under stress and different light spectra from control plants were shown (Fig. 6).

According to ANOVA results (Table 3), stress, light, and their interaction effects changed the JIP-test parameters significantly. Salinity and alkalinity stress induced an increase in F_O_ and decreased F_M_ and, as a result, a decrease in F_V_ and area. In salt-treated plants, blue light caused a significant rise in F_M_, F_V_, and area parameters. Red light had the most significant effect on reducing the F_O_ parameter. Under alkaline stress, red light had the most significant effect on increasing F_M_, F_V_, and area parameters. The performance indexes (PI_ABS_ and PI_total_) were significantly affected by treatments. Both stress treatments caused a significant decrease in these parameters and showed that performance index parameters are susceptible to stress conditions. Under salinity stress, red and white/yellow light had the greatest effect on PI_ABS_ and PI_total_ parameters, respectively. Under alkaline stress, using red and blue/red light spectra significantly increased these parameters compared to without complementary light treatment. Salinity and alkalinity stress significantly reduced Quantum yield parameters (φ_PO_, φ_EO_, φ_RO_, Ψ_EO_, δ_RO_). The blue, red, and their combination spectra reduced the effects of stress and increased the quantum performance parameters compared to the without supplementary light treatment. Both stress treatments affected reaction centers, and ABS/RC, DI_O_/RC, and TR_O_/RC parameters increased. ET_O_/RC and especially RE_O_/RC decreased under stress conditions, and salinity stress had a more significant effect on these parameters. Under salinity stress, blue light spectra had a significant effect on the RE_O_/RC parameter. Under alkaline stress, blue/red light spectra had the greatest effect on ET_O_/RC and RE_O_/RC parameters. The V_J_, d_VG_/dt_O_, and d_V_/dt_O_ parameters increased in the plants treated with NaCl and NaHCO_3_. Different light spectra reduced the effects of stress on these parameters compared to treatment without complementary light (Fig. 6).

**Table 3:** ANOVA results of different light spectra and stress on JIP-test parameters in strawberry cv. Paros.

Source of variations	Means square											
	Area	F_O_	F_M_	Fv	F_O_/F_M_	F_V_/F_M_	F_V_/F_O_	Vj	dVG/dto	dV/dto	Vi	Sm
Light (L)	***	***	***	***	***	***	***	***	***	***	***	*** *** ***
Stress (S)	***	**	***	***	***	***	***	***	***	***	***	
L × S	***	***	***	***	***	***	***	***	**	***	**	

Source of variations	Means square											
	ABS/RC	Dio/RC	TRo/RC	Eto/RC	REo/RC	Φ(po)	Ψ(Eo)	Φ(Eo)	δ_(RO_)	Φ_(RO)_	PI_ABS_	PI_total_
Light (L)	***	***	***	**	***	***	***	***	***	***	***	*** *** ***
Stress (S)	***	***	***	**	***	***	***	***	***	***	***	
L × S	***	***	***	**	***	**	***	***	***	***	***	

*, **, ***Significance at the 0.05, 0.01, and 0.001 probability levels, respectively, ns, not significant. SAS software version 9.4 was used for data analysis (https://www.sas.com/en_us/home.Html).

### Correlation analysis

**Figure 7:**
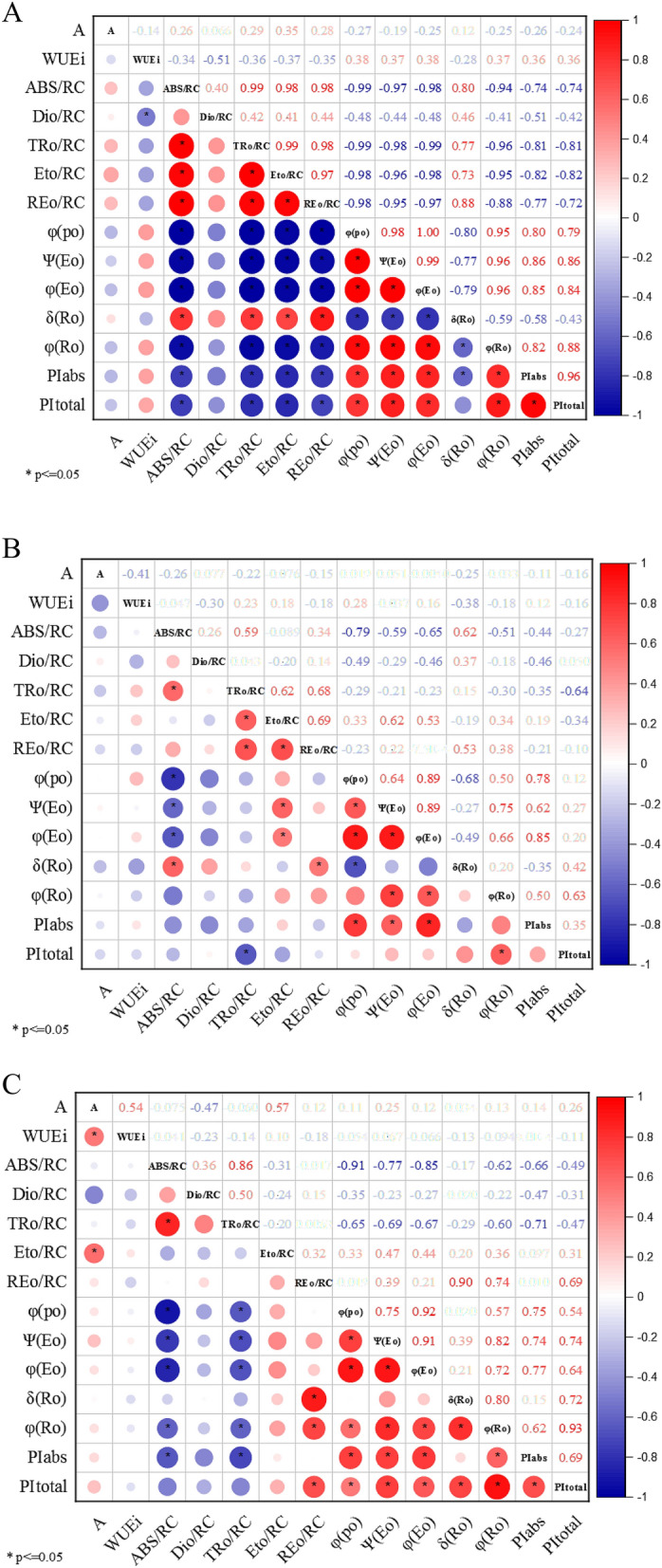
Correlation plot between the plant gas exchange (**A**, WUE_i_) and prompt fluorescence parameters. (**A**) In control conditions (non-stressed plants); (**B**) under salt stress; (**C**) under alkalinity stress. The size and color intensity of circles are proportional to Pearson’s correlation coefficient at p < 0.01. Red circles indicate positive correlations, while blue are negative correlations. In the correlogram scale from −1 to + 1, Pearson’s correlation coefficient for variables is on the vertical and horizontal axis. Asterisk indicates values that are statistically different at p < 0.05. The correlation plot was drawn with Origin Pro software version 2021 (https://www.originlab.com/2021).

Based on the statistical analysis, it was observed that there is a significant correlation between some chlorophyll and fluorescence and the CO_2_ assimilation parameter (A). Figure 7 shows the correlations between the CO_2_ assimilation parameter (A) and the fluorescence chlorophyll parameters (ABS/RC, TRo/RC, Eto/RC, REo/RC, PI_ABS_, and PI_total_). The CO_2_ assimilation parameter (A) correlated positively with Eto/RC, REo/RC, PI_ABS_, and PI_total_.

### Principal component analysis

**Figure 8:**
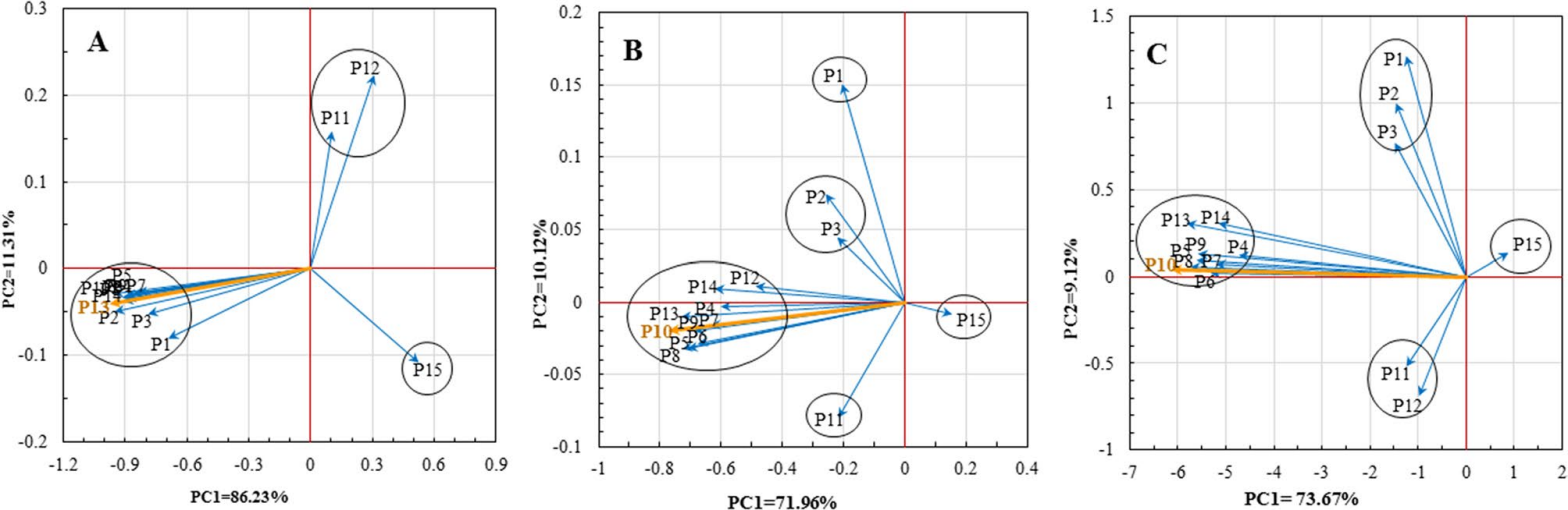
Principle component biplot of JIP test parameters based on variations of 5 levels of light spectra under (**A**) control (non-stress); (**B**) salinity stress; (**C**) alkalinity stress. P1: ABS/RC; P2: Dio/RC; P3: TRo/RC; P4: Eto/RC; P5: REo/RC; P6: φP_O_; P7: ΨE_O_; P8: φE_O_; P9: δR_O_; P10: φR_O_; P11: PI_ABS_; P12: PI_total_; P13: V_j_; P14: V_i_; P15: S_m_. Principal component analysis was performed using MATLAB software version R2015b (https://www.mathworks.com).

First, the data were standardized to zero mean and unit variance. PCA was performed to summarize the variations of 15 parameters during five-light spectra treatments at the control treatment and the two stress levels, separately. In the control treatment, the performed PCA explained 97/54% of total variations of five light spectra treatments (Fig. 8A). This value was 82.08 and 82.79% for salinity and alkalinity treatments, respectively (Fig. 6B,C). Most of the variations were explained by the first component. Thus, the effect of five light spectra treatments on the variations of the 15 parameters was linear. Based on two principal components, the 15 evaluated parameters at the control treatment were divided into three groups, and the salinity and alkalinity stress treatments into five and four separate groups, respectively (Fig. 8). Regardless of the direction of the effect, the parameters p11, p12, and p15, respectively, had the least contribution to the first principal component of the variations caused by five light spectra treatments at the control treatment. These parameters were p15, p1, p11, p3, and p2 at salinity stress treatment, and alkalinity stress treatment were p15, p12, p1, p2, p3, and p11. The other parameters have the most contribution to the first principal component in control and two stress treatments. Among these parameters, at the control treatment, parameter p13, and two stress treatments, parameter p10 had the most contribution in the first principal component. Thus, in studying the effect of light spectra treatments on various parameters, it is necessary to pay special attention to these two parameters.

## Discussion

The use of LED as a light source is important in terms of improving plant productivity because not only light intensity but also its spectral composition affects plant life processes. Therefore, in this work, we compared four different light spectra in terms of photosynthesis performance and chlorophyll fluorescence parameters of strawberry cv. Paros under salinity and alkalinity stress conditions.

Inhibition of photosynthesis in saline and alkaline stresses on different plant species was investigated^27,28^. In this experiment, we investigate the effect of complementary light spectra on increasing the tolerance of strawberry plants to salinity and alkalinity stress. The oxygen-evolving complex (OEC) was described by Schreiber et al.^29^ as one of the most sensitive components of the electron transfer chain of the photosynthetic apparatus. Usually, its decreased efficiency is caused by an electron transport disorder. Our results showed that under salinity and alkalinity stress, the absorption and trapping of electrons decreased. Based on these results, the amount of ABS/RC and TRO/RC increased in both stresses, which is due to the loss of active reaction centers. Under salinity conditions, due to the dissociation of LHCII and PSII, the efficiency of trapping electrons in the PSII reaction center decreases^30^. Based on this knowledge, there is known that photosynthesis is restrained by both stresses. A study of alkalinity and salinity stress showed an additional impact of alkalinity stress due to increased pH. The high pH condition around the roots will precipitate metal ions, P, and Mg precipitation is caused by alkalinity stress, leading to inhibition of chlorophyll synthesis^31^. Photosystem II (PSII) has also been shown to play a major role in the reaction of photosynthesis to stress conditions^32,33^, which was confirmed by these studies. Our results showed that the maximum quantum yield of primary PSII photochemistry (φPo) decreased under the influence of both stresses. In tomato seedlings, PSII electron transfer is blocked by salinity-alkalinity stress^34^. PSII activity decreases due to the harmful effects of salinity stress on the Mn cluster, and PSI activity decreases due to plastocyanin/cytochrome c553 separation^35^. Electron transport inhibition of PSII could contribute to ROS production, such as hydrogen peroxide (H_2_O_2_), hydroxyl ions, and singlet oxygen (^1^O_2_)^36^, which could destroy the protein D_1_^37^. Finally, stresses reduce performance indexes (PI_ABS_ and P_Itotal_). Under stress conditions, photosynthetic parameters are significantly affected, and this indicates that the photosynthetic apparatus is vulnerable to stress^38^. Our studies confirmed that there are effects on chlorophyll fluorescence in both stresses.

The stress of salinity and alkalinity affects the amplitude of both the F_O_, and F_M_ parameters. F_O_ increases and F_M_ decreases, indicating no electron transfer from P_680_ to Q^A-^ and energy dissipation of PSII excited states^39^. The results showed that both salinity and alkalinity stress increased the energy dissipation of PSII, but salinity stress had a greater effect on energy dissipation. The rise in the initial level of fluorescence (F_O_) observed could be attributed to the increase in the slope of the initial resulting from the K-step^40^. Our results (Fig. 4) showed that alkalinity stress caused a significant rise in F_O_ compared to salinity stress with an increasing K band. A significant delay of F_M_ can indicate inhibition of electron flow by PSII. It can result from non-photochemical quenching, D_1_ protein degradation, or PSII RC inactivation^17^. In salt-stressed wheat leaves, PI_ABS_ decreased due to ionic and osmotic stress^15^, and the decline of PI_ABS_ was associated with a decrease in (F_M_ – F_J_)/F_V_^41^. A reduction in the maximum quantum yield of PSII (φ_PO_) shows that the stresses inhibit the redox reaction after Q^A−^ and slow down the electron transition between Q^A−^ and Q^B^ (Fig. 6). The decreased outflow of electrons on the acceptor side of PSI due to the inactivation of ferredoxin NADP^+^-reductase was shown by a lower δRo level^42^. Alkalinity stress in strawberry plants has been reported to decrease PI and S_m_^20^; similar results have been reported under mixed salinity-alkaline stress conditions by Deng et al.^43^. The total number of photons absorbed from all reaction centers is expressed by chlorophyll molecules divided by the total number of active reaction centers by ABS/RC. It is affected by the ratio of active/inactive RCs, and the ABS/RC ratio also increased as the number of inactive centers increased^44^, which decreases the transport of electrons in active RC (ET_O_/RC) and reduces the final acceptor in PSI (RE_O_/RC). Plants were grown in blue and blue/red light, electron transport flux per reaction center, and the probability that the trapped exciton would transfer the electron in the electron transport system beyond Q^A−^ was increased following the stress condition (Fig. 8). This indicates that plants grown in blue and blue/red light were more capable of carrying electrons from absorbed photons through the electron transport chain and beyond Q^A−^. This indicated that the energy level in reaction centers was positively regulated by plants grown in blue and blue/red light^45^ following stress exposure.

In our research, the L band was affected by stress conditions and light spectra. Alkalinity stress significantly increased the L and K bands. In the treatment of blue and red spectra, L and K bands were at a lower level than other light spectra, Which indicates the effect of these spectra on the stability and structure of reaction centers and PSII antennas^8^. Inactivation or inhibition of electron transfer on the donor or acceptor side of PSII by the oxygen-evolving complex (OEC) may be associated with the K band in the transient OJIP^46^. It was indicated that the "K-step" could be correlated with irreversible stress damage in PSII^40^.

According to studies, changes in the spectrum of light have a significant impact on the physiological processes of the strawberry plant. These conclusions were confirmed by Hogewoning et al.^47^ or Macedo et al.^48^. We also found that under salinity and alkalinity stress, blue and blue/red light had a positive effect on CO_2_ assimilation. Salinity and alkalinity stress reduced water use efficiency (WUE_i_) compared to the control. The blue/red light had a significant effect on WUE_i_ and increased it in all stress conditions. For chlorophyll synthesis, blue light is essential, but red light is also involved in this process^47^. Plants absorb blue and red light, mostly (about 90%). Blue light leads to an increase in stomata dilation and, consequently, to an increase in transpiration, and red light inhibits stomata dilation, which leads to a reduction in transpiration^49^. The response of stomatal guard cells to a decrease in intercellular CO_2_ concentration and the direct reaction of guard cell chloroplasts to red light leads to red light-induced stomatal opening^50^. Blue light is mainly used in commercial research and horticulture because it is one of the most important spectra in the process of photosynthesis. At the same time, the lack of one of them (red or blue light) reduces the efficiency of photosynthesis.

Photosynthesis is impaired by salinity by stomatal closure, photochemical reaction destruction, and carbon assimilation. The stomatal closure is the first defense of the plant against salinity–alkalinity stress^51^. Due to the increased resistance of diffusion in the pores and through mesophilic cells, the supply of CO_2_ in stressful environments decreases^52^. The reduction of CO_2_ disrupts the electron transfer chain. In the isolated epidermis, it is accepted that red light causes stomatal opening. This reaction depends on the chloroplasts of the guard cells^53^. Both C_i_ and guard cell chloroplasts play a role in the synergistic effect of blue and red light on stomatal opening. The blue-light reaction can be indirectly induced by Mesophyll photosynthesis^54^. For blue-light signaling in guard cells, Ca^2+^ was indicated to be responsible. Both blue-light-dependent H^+^ pumping and stomatal opening were inhibited by calmodulin antagonists^55^.

A good correlation was observed between the increased capacity to assimilate CO_2_ and the increased value of PI_total_, PI_ABS_, Eto/RC, and REo/RC. The finding provided evidence of the correlation of OJIP fluorescence variations with changes in general photosynthesis capacity during stress in field or laboratory conditions. The coordinated control of the entire photosynthesis system takes place in such a way as to maintain an internal balance between the efficiency of the photosynthetic light phase reaction and the efficiency of the reactions leading to CO_2_ assimilation^56^.

## Conclusions

From our studies, it can be concluded that the photosynthesis apparatus of strawberry plants are sensitive to both types of stressors. The influences of the adverse conditions could be mitigated by proper choice of lighting (quality). Under salt and alkalinity stress, blue/red and blue light had a significant effect on the assimilation of CO_2_ and photosynthetic electron transfer chains. Using blue and blue/red light as complementary light in the greenhouse can increase the tolerance of plants to salinity and alkalinity stresses. Chlorophyll fluorescence measurements were a reliable tool for monitoring and early detection of the changes caused by the stressor. Previous studies have mainly focused on the effect of light intensity on plant growth and development. However, there is limited information on the effects of different light spectra on plant photosynthetic efficiency and plant gas exchange under stress conditions. The intensity and quality of supplemental light for plants vary under greenhouse conditions. It is suggested that this research be done other plants and cultivars and in different spectra and intensities of light under stress conditions. This research will be helpful for improving the growth and development of plants in greenhouses and under stress conditions. In greenhouse crops and under abiotic stresses, it is possible to enhance the growth and development of plants by applying complementary light spectra.

## Supplementary Information

Supplementary Information 1.

Supplementary Information 2.

Supplementary Information 3.

## Data Availability

All data generated or analyzed during this study are included in this published article (and its supplementary information files).
