# Supplementary material for: The effect of supplementary light on the photosynthetic apparatus of strawberry plants under salinity and alkalinity stress
Source: Sci Rep. 2022 Aug 2;12:13257. doi: 10.1038/s41598-022-17377-8 (PMC9345948; doi:10.1038/s41598-022-17377-8)
Supplement: Supplementary file 2 — Supplementary Information 2. [file 41598_2022_17377_MOESM2_ESM.docx]

1. Artificial light and special light spectra in the greenhouse under stress conditions can be used to investigate the effects of specific spectra on plant tolerance under stress conditions.
2. Influences of salt and alkaline stresses can be mitigated by properly selected lighting.
3. Under salinity and alkalinity stress, blue/red light had the positively effect on photosynthesis process.
4. JIP test parameters were significantly affected by different light spectra.
